# Targeting DNA Methylation in the Adult Brain through Diet

**DOI:** 10.3390/nu13113979

**Published:** 2021-11-08

**Authors:** Joseph Allison, Aleksandra Kaliszewska, Sara Uceda, Manuel Reiriz, Natalia Arias

**Affiliations:** 1 Department of Basic and Clinical Neuroscience, Institute of Psychiatry, Psychology and Neuroscience, King’s College London, Denmark Hill, London SE5 8AF, UK; joseph.c.allison@kcl.ac.uk (J.A.); aleksandra.kaliszewska@kcl.ac.uk (A.K.); 2 BRABE Group, Department of Psychology, Faculty of Life and Natural Sciences, University of Nebrija, C/del Hostal, 28248 Madrid, Spain; suceda@nebrija.es (S.U.); mreiriz@nebrija.es (M.R.); 3 Institute of Neurosciences of the Principality of Asturias (INEUROPA), 33003 Oviedo, Spain; 4 Health Research Institute of the Principality of Asturias—ISPA, 33011 Oviedo, Spain

**Keywords:** DNA methylation, brain, microbiota, nutrition, cognition, SAM, epigenetics

## Abstract

Metabolism and nutrition have a significant role in epigenetic modifications such as DNA methylation, which can influence gene expression. Recently, it has been suggested that bioactive nutrients and gut microbiota can alter DNA methylation in the central nervous system (CNS) through the gut–brain axis, playing a crucial role in modulating CNS functions and, finally, behavior. Here, we will focus on the effect of metabolic signals in shaping brain DNA methylation during adulthood. We will provide an overview of potential interactions among diet, gastrointestinal microbiome and epigenetic alterations on brain methylation and behavior. In addition, the impact of different diet challenges on cytosine methylation dynamics in the adult brain will be discussed. Finally, we will explore new ways to modulate DNA hydroxymethylation, which is particularly abundant in neural tissue, through diet.

## 1. Introduction

**Figure 1:**
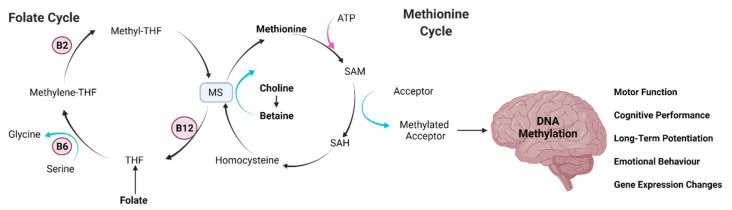
Key dietary molecules that modulate DNA methylation and their link to cognitive changes. The overview shows the interplay between the folate cycle and the methionine cycle, which produce S-adenosyl methionine (SAM) in order to methylate DNA within the brain. Brain DNA methylation has led to cognitive and genetic changes that ultimately could drive to neurodegenerative processes. Abbreviations: adenosine triphosphate, ATP; methionine synthase, MS; S-adenosyl homocysteine, SAH; tetrahydrafolic acid, THF.

DNA methylation is a major epigenetic mechanism involving direct chemical modification to DNA. DNA methylation is catalyzed by a family of DNA methyltransferases (DNMTs) that transfer a methyl group from the methyl donor S-adenosyl methionine (SAM) onto the 5′ carbon position of the cytosine ring residing, in most cases, at the dinucleotide sequence CG [1,2,3,4]. Among all the DNMTs, DNMT1, known as a “maintenance” methyltransferase, has a preference for hemimethylated substrates and is involved in copying DNA methylation patterns from the parental DNA strand onto newly synthesized daughter strands. DNMT3a and DNMT3b can establish new methylation patterns to unmodified DNA and are thus known as “de novo” DNMTs. Their function suggests a role during cellular replication [5] and it was assumed that, by the time cells reach terminal differentiation, DNMT expression is much reduced. This would seem to suggest that the DNA methylation pattern in postmitotic cells is stable. However, postmitotic neurons in the mature mammalian brain still express substantial levels of DNMTs, raising the possibility that DNMTs and DNA methylation may play a novel role in the brain (overviewed in Figure 1) [6,7].

In this regard, Numata et al., (2012) has proved the role of epigenetics in cortical development across the lifespan by examining the prefrontal cortex in a genome-wide study of DNA methylation in ~14,500 genes at ~27,000 CpG loci focused on 50 promoter regions in post-mortem samples from 108 individuals aged from 14 weeks of gestation to 83 years [8]. They demonstrated that DNA methylation shows unique temporal patterns across the lifespan, with the fastest changes occurring prenatally, which then slow down with ageing. This leads to the possibility that prevention or reduction in epigenetic drift could alleviate disorders and diseases associated with ageing. In this respect, findings from post-mortem human brains demonstrate the involvement of DNA methylation and hydroxymethylation in Alzheimer’s disease, emphasizing the importance of the timing of epigenetic changes during the progression of pathology [9]. Moreover, ageing in the hippocampus, a key brain area in several cognitive functions, has shown changes in DNA methylation and hydroxymethylation, DNMT3a and histone deacetylase (HDAC) [9,10].

Another important characteristic of DNA methylation in vertebrates is the cell-specific pattern of the distribution of methylation on CG dinucleotides [11]. DNA methylation in critical sites silences genes by two principal mechanisms. First, methylation in critical sites inhibits the binding of transcription factors to their recognition elements [12,13]. Second, the methylation of a regulatory region of DNA recruits methylated DNA binding proteins such as MeCP2, which is a methyl binding protein expressed in neural precursors and stem cells, and chromatin modification enzymes such as HDACs to the gene [14,15,16,17] which, in turn, introduce histone modifications resulting in the silencing of chromatin.

MeCP2 has been broadly studied in the central nervous system (CNS), not only because of its role in Rett syndrome and some forms of mental retardation [18], but also due to the fact that neural activity leads to phosphorylation of MeCP2, altering its ability to bind gene promoters and silence gene expression [19,20]. MeCP2 is required for normal neuronal maturation, and its loss or the loss of its ability to be phosphorylated results in aberrant dendritic arborization, synaptic function, and plasticity with learning and memory deficits [21,22,23,24,25,26,27]. Another study from Lubin et al., (2008) [28] observed that when neuronal activity is inhibited during fear conditioning, memory formation and changes in DNA methylation are prevented. All these results suggest that DNA methylation patterns are dynamic in neurons (also read [29,30,31]) and respond to cellular signaling pathways, which provide a link between the extracellular environment and the state of DNA methylation. That is, signaling pathways that activate chromatin-modifying enzymes could potentially result in altering DNA methylation patterns.

### 1.1. The Role of Diet in DNA Methylation

As has been previously discussed, epigenetic marks react dynamically to external signals and can be modified based on the availability of cofactors and metabolites during adulthood. Indeed, changes in nutrition can modulate DNA methylation in the adult CNS. In line with this, feeding post-weaning mice with a diet deficient in folate, methionine and choline caused promoter hypermethylation and low expression of specific subunits of AMPA (amino-3-hydroxy-5-methyl-4-isoxazolepropionic acid) receptors, together with impaired hippocampal-dependent memory [32]. Also, a methyl donor supplementation diet in adulthood was able to rescue depression-like behavior induced by maternal separation, and increased global brain DNA methylation in adult rats [33]. Moreover, a high fat diet increased DNA methylation and downregulated dopamine 2 receptor in the striatum in experimental animals, whereas administration of oryzanol, the bioactive constituent of brown rice, decreased the expression and activity of DNMTs and restored the level of the receptor [34]. Another study has shown that obesity induced by diet provoked hypermethylation in all the promoters of the memory-related genes such as Ppp1cb, ReIn and Sirt1 which was accompanied by impaired hippocampal performance in spatial memory and synaptic plasticity [35].

Finally, diet modifications such as caloric restriction have shown improved cognition, neurogenesis and, more importantly, were able to induce a de novo pattern of CG and CH methylation in the aging brain [36]. This evidence suggests that nutrition and aging are profoundly intertwined therefore, in this review we will discuss how manipulations of the diet could modulate DNA methylation in the adult brain.

### 1.2. The Microbiome as a Modulator of DNA Methylation

Another underestimated contributor to the topic of diet-driven neuroepigenetics in adulthood is the host’s gut microbiota. Indeed, the intestinal microbiota work as a factory to produce complementary endogenous sources of, among others, vitamin B12, B6 and folate, as well as contributing to energy harvesting from food and nutrient absorption [37]. Thus, the host’s microbiome could potentially modulate the availability of methyl donors for DNA methylation in the CNS and therefore impact behavior.

In this respect, it has been shown that microbial-derived short-chain fatty acids (SCFA), such as acetate, butyrate and propionate obtained through the fermentation of indigestible polysaccharide in the diet, work as HDAC inhibitors, and have been shown to cross the blood–brain barrier. It is well known that SCFAs contribute to the maturation and maintenance of microglial cell function [38], and may modulate the levels of neurotransmitters and neurotrophic factors. In addition, altered levels of SCFAs have been observed in several brain disorders [39] and can influence DNA methylation in colon cancer cells [40]. In this review, we will discuss the influence of gut microbiota on brain DNA methylation.

## 2. Role of Microbiota in Regulating This Relationship between Diet, DNA Methylation and Cognition in the Adulthood

### 2.1. The Symbiotic Association between Intestinal Microbiota and Their Host

The human microbiota consists of approximately 10–100 trillion microorganisms which reside on and within our bodies [41]. Between 500–1000 different species of bacteria are estimated to inhabit an average person, with the colon being the most abundantly populated part of the body [42,43]. The majority of bacteria found in the gut belong to either one of just two phyla: Bacteroidetes or Firmicutes [44]. Microbial cells outnumber our own cells tenfold, and the genes they harbour, known as the microbiome, may represent as much as 90% of our genetic material [43,45]. In return for providing the microbes with a nutritionally rich and shielded environment to live in, humans rely on microbiota to carry out essential metabolic functions such as the breakdown of otherwise indigestible complex carbohydrates and the synthesis of bioactive compounds [37,46]. In fact, microbial metabolism of food matter in the digestive track is our only source of multiple vital metabolites, some of which are known to have neuromodulatory effects [47,48,49,50]. A compelling body of evidence suggests that gut health is essential for the proper development and functioning of bodily systems, including the CNS. Concordantly, reduction in microbial diversity and loss of homeostasis within the microflora of the gut, or dysbiosis, have been associated with a number of neurological disorders, including cognitive impairment and dementia [51,52,53,54,55]. Thus, the relationship between human hosts and their microbiota affects a multitude of physiological processes and has critical consequences for human behaviour, including cognition.

### 2.2. The Microbiota–Gut–Brain Axis as a Modulator of Cognitive Behaviour

The intestinal microbiota interacts with the brain to elicit extra-intestinal effects via the gut–brain axis, a bidirectional line of communication consisting of neuronal, neuroimmune, endocrine and metabolic signals [56]. In regards to neuronal pathways, the vagus nerve (VN) acts as a link between the gut and the brain by providing a physical connection from the enteric nervous system (ENS) to the CNS [57]. Although the precise mechanisms by which the brain and microbiota are functionally linked remain to be elucidated, we know that the brain can regulate gastrointestinal functions by triggering the release of signalling molecules from intestinal cells into the gut lumen [58]. In turn, microbe-derived metabolites such as neurotransmitters, hormones, choline metabolites, vitamins and SCFAs can both directly and indirectly influence the CNS physiology.

Chemoreceptors located on the vagus nerve can sense and react to compounds of microbial origin [59], with SCFAs such as butyrate shown to directly activate the afferent fibres on the VN [60]. In vivo manipulation of the intestinal microbial composition via an intraduodenal injection of *Lactobacillus johnsonii* was reported to stimulate the gastric activity of the VN [61]. Moreover, long-term oral administration of Lactobacillus bacteria *L. rhamnosus* in mice induced brain region-dependent alterations in the expression of gamma aminobutyric acid (GABA) receptors, i.e., the main inhibitory neurotransmitter in the CNS [62]. Multiple brain regions with important roles in learning, memory formation, emotional behaviours and fear conditioning, including the hippocampus, cingulate and amygdala [63], were affected. Supplementation with *L. rhamnosus* was found to render animals more resilient against stress paradigms used to induce depressive and anxiety-like behaviours. Interestingly, the beneficial effects of *L. rhamnosus* were not observed in vagotomised mice, thus suggesting that an intact vagus nerve connecting the intestinal microbiota and CNS is essential for the behavioural manifestations of dietary interventions with probiotics like *L. rhamnosus*. Similarly, depletion of acetate-producing bacteria following antibiotic treatment with vancomycin reduced hippocampal levels of synaptophysin, a marker of plasticity implicated in cognition [64], and induced learning and memory deficits in a mouse model of diabetes [65]. The cognitive impairment and neurochemical changes were reversed following exogenous supplementation with SCFA acetate or microbial recolonization via fecal microbiota transplant. However, this effect was also attenuated by vagal inhibition.

Microbial regulation of cognitive behaviour has been shown to be mediated not only by pathways originating within the ENS, but also through direct interactions between the CNS and microbe-derived metabolites. A recent study investigating the role of microbiota in the development of mouse brains and behaviour has shown that metabolites of exclusively microbial origin, as well as those produced as a result of host-microbiota combinatorial metabolism, can cross the blood–brain barrier (BBB) [66]. The BBB acts as a protective barrier, restricting the entry of molecules from systemic circulation into the brain; therefore only compounds capable of crossing the BBB may gain access to the brain and elicit biological effects by binding to neuronal receptors. One of the microbiota-derived metabolites detected in the forebrain of those animals [66], imidazole propionate, is synthesised exclusively from the bacterial metabolism of dietary histidine and is known to affect host signalling by activating mechanistic target rapamycin complex 1 (mTORC1) [67]. Given that mTORC1 is implicated in neurogenesis, synaptic formation and its knock down or inhibition leads to deficits in learning and memory [68], the presence of microbiota-derived imidazole propionate in the brain where it can interact with mTORC1 has important implications for the microbial regulation of human behaviour. Indeed, imidazole propionate levels were found to be associated with cognitive performance in human patients [69].

### 2.3. Microbiota-Derived Metabolites in Host Epigenetic Regulation

Bioactive compounds produced by microbiota can also shape human behaviour by acting as epigenetic regulators and can modulate the expression of genes involved in cognition. Dysbiosis and complete absence of intestinal microbiota have been shown to induce potentially long-lasting epigenetic modifications in human subjects and animal models, and have also been associated with behavioural alterations.

Studies comparing germ free (GF) mice, which lack microbiota, with control animals have reported changes in histone methylation, disruption of transcription profile of synaptic plasticity genes in the hippocampus, and deficits in learning and memory [70,71,72,73,74]. Some of those changes were shown to be reversed following dietary supplementation with SCFAs such as butyrate or re-colonisation with normal microbiota. A study investigating behavioural phenotypes, epigenetic profiles and microbial composition in mouse models of Alzheimer’s disease (AD) found a genotype-dependent association between microbiota, and more specifically the Lachnospiraceae and Ruminococcaceae families of bacteria, and cognitive performance in the animals [75]. Additionally, a relationship between microbiota and DNA methylation in the hippocampus was reported, thus drawing a link between alterations in microbiota, DNA methylation status and cognition in this model of neurodegenerative disease. Another study investigating the behavioural and epigenetic outcomes following administration of probiotics in Zebrafish reported changes in DNA methylation of the brain-derived neurotrophic factor (BDNF) and tryptophan 5-monooxygenase (Tph1A) genes [76] which are implicated in behaviour, mood and cognition in humans [77,78]. Whilst the effects of human microbial composition on cognitive performance [79] and the role of epigenetics in cognition [80] have been studied, few studies explore the link between DNA methylation and the microbiome in relation to human cognition. However, in a group of 20 obese human subjects, an association between global DNA methylation pattern and gut microbiota composition, or more specifically the ratio of Bacteroidetes to Firmicutes, was found [81]. High abundance of Firmicutes was also associated with hypermethylation of 568 genes in another human subject study [82]. Interestingly, a shift in the abundance of Firmicutes and Proteobacteria was linked to AD in human subjects with reduced faecal abundance of Firmicutes, correlating with the severity of symptoms in these patients [83].

The two main ways by which microbial metabolites are known to induce changes in the human epigenome are i) direct suppression or recruitment of the enzymes mediating methylation, and ii) altering the availability of essential substrates and cofactors required by epigenetic enzymes [84]. The key mediators of microbial-dependent epigenetic alteration in the human brain are SCFAs [85]. These bioactive compounds: butyrate, acetate, propionate and folate, to mention just a few, are produced exclusively as a result of intestinal microbiota-dependent anaerobic fermentation of complex dietary carbohydrates [86,87,88]. The levels at which they are expressed are regulated by the host microbial composition in a diet-dependent manner [89]. The main substrates for the bacterial production of SCFAs derived from non-digestible dietary fibre are: resistant starch, inulin, oat bran, wheat bran and cellulose [90]. The intake of dietary-resistant starch in particular is associated with abundance of butyrate, as it is an essential substrate for its synthesis [91]. Dietary intervention using oat bran was found to enhance microbial synthesis of butyrate in a human population of colitis [92]. Under healthy physiological conditions, the three most abundant SCFAs: acetate, propionate and butyrate, are synthesised in the ratio of 60:20:20, with the combined concentration of the three reaching approximately 100mM in the human gut lumen, with much lower levels detected in systemic circulation [93]. Notably, the ratio in which Bacteroidetes and Firmicutes are expressed in the gut has important implications for the abundance of individual SCFAs as bacteria from Bacteroidetes phyla mainly synthesise acetate and propionate, whereas Firmicutes produce mainly butyrate [86]. Given that the ratio of Bacteroidetes to Firmicutes is linked to human cognition and cognitive deficits in AD, it is possible that the changes in SCFAs expression mediate these effects.

Moreover, certain strains of bacteria, such as *Bidobacterium* and *Lactobacillus plantarum* are known to produce folate in the gut [94]. Folate is a methyl donor required for the generation of SAM, the primary methyl donating substrate of DNMTs, and is therefore directly involved in DNA methylation [95]. Folate deficiency has been linked to cognitive impairments, which are believed to be mediated by altered gene expression patterns, especially in the hippocampus [96]. Similarly, microbial metabolism of the B vitamins riboflavin (B2) and biotin (B8), which participate in one-carbon metabolism, provide methyl donor substrates for SAM, thus facilitating DNA methylation processes [97,98]. Dietary riboflavin supplementation was found to improve global cognitive function in middle aged and elderly human subjects, although the contribution of microbiota compositional changes was not assessed in this study [99]. On the other hand, microbial consumption of choline reduces the bioavailability of this essential nutrient, which also acts as a methyl donor. Overexpression of choline-utilizing bacteria, the *E. coli* MS 200-1 strain belonging to the family of Enterobacteriaceae, was associated with global changes in DNA methylation profiles in multiple tissues including the brain, increased susceptibility to metabolic disease and aberrant behaviours reminiscent of choline deficiency including deficits in learning and memory in mice [100].

### 2.4. Interaction between Diet, Microbiota and Epigenetics in the Adult Brain

Diet is arguably the most influential lifestyle determinant of intestinal microbiota composition. Specific dietary habits such as long-term adherence to a vegan/vegetarian diet, or the consumption of Western vs. Mediterranean diets, are associated with distinct microbial profiles [101,102,103]. Even transient dietary changes can induce alterations in the abundance of various bacterial strains and shift the ratio of commensal/pathogenic microorganisms [104,105]. As discussed in the previous section, the composition of intestinal microbiota determines the production of bioactive metabolites which in turn regulate DNA methylation patterns in the adult brain and affect cognitive performance. Therefore, any changes in dietary habits which induce alterations in the composition of intestinal microbiota may affect host epigenetics. The complex interaction occurring between dietary substrates, microbiome and epigenetics are referred to as the microbiota–nutrient metabolism–host epigenetic axis [106].

Given the importance of SCFAs in microbiota-dependent epigenetic modifications, sufficient intake of fibre is crucial. A study comparing the microbiome of children from Burkina Faso and Europe found that a high-fibre diet consumed by children from Burkina Faso resulted in greater microbial diversity, higher abundance of beneficial bacteria Prevotella and Bacteroides, and higher levels of SCFA butyrate than their European counterparts consuming Western diets, which are much lower in plant-derived fibre [107]. The phytochemicals contained in high-fibre foods are metabolised by gut microbiota not only into SCFAs but also polyphenolic derivatives and isothiocyanates [108], both of which are implicated in the epigenetic regulation of gene expression [109,110]. The interaction between polyphenols and microbiota is two-fold. On one hand, polyphenols act as probiotics in shaping the intestinal microbiota, with the changes they elicit depending on the specific polyphenolic compound and the overall diet followed [111]. Whilst on the other hand, polyphenolic compounds also undergo microbial metabolism. The polyphenols contained within green tea, green tea catechins, have been shown to inhibit the activity of DNMTs, resulting in re-expression of the genes involved in WNT-signalling (wingless/int), among others, in vitro [112]. Catechins are converted in a microbiota-dependent manner into phenolic acids, which, when assessed in an in vitro enzymatic assay, were found to significantly reduce the activity of DNMTs [113,114]. In vivo, supplementation with a standardized bioavailable polyphenolic preparation was found to alter gene expression in the hippocampus of mice, as well as the activity of DNMTs [115].

Furthermore, the majority of key compounds implicated in the one-carbon metabolism are not only microbial metabolism-dependent, but also diet-dependent and susceptible to dietary interventions. For example, even though the microbial synthesis of folate was reported to be significant, given the fact that folate deficiency was observed following antibiotic treatments [116,117], green leafy vegetables, nuts and pulses are all dietary source of folate [118]. A longitudinal study enrolling 2533 participants has found adequately sufficient intake of folate, as well as vitamin B12, B6 to be significantly associated with improved cognitive performance, whereas deficiency in dietary intake of B12 was linked to accelerated cognitive decline [119]. Additionally, in a case-control analysis of DNA methylation conducted as part of this study, individuals presenting with mild cognitive impairment were found to have increased levels of the methylation of redox-related genes.

## 3. Impact of Diet on DNA Methylation on Adult Brain

**Figure 2:**
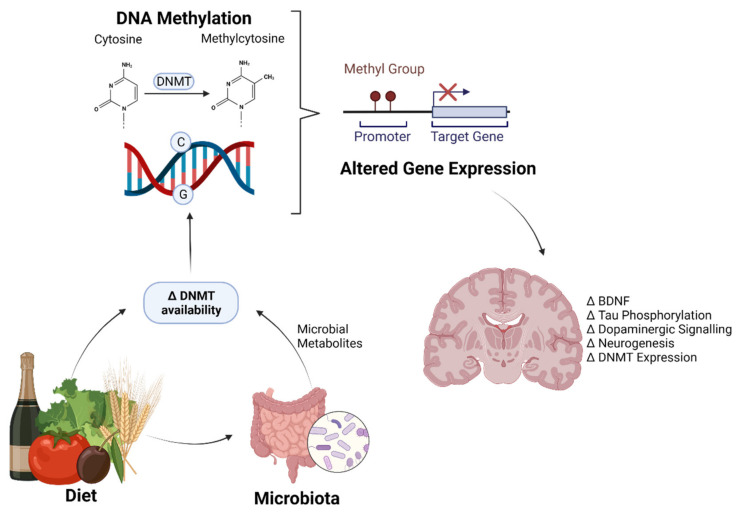
Diet and the gut microbiome regulate brain gene expression through epigenetics. An overview of how the diet and gut microbiome alter DNMT (DNA methyltransferase) availability, either through loss of cofactors/one-carbon molecules, or inhibition of DNMTs. This alters the methylation status of cytosine residues in DNA, which, when in promotor regions of genes, can prevent transcription. Genes commonly altered by the diet and microbiome that affect cognition include BDNF (brain derived neurotrophic factor), Tau, dopamine-related genes, neurogenesis-related genes and DNMTs themselves. Created with BioRender.com.

Diet is one of the largest manipulable factors when it comes to DNA methylation. Malnutrition is classified as either under- and overnutrition, resulting in almost 2.3 billion people being classified as malnourished just on the basis of their weight [120]. This is disregarding individuals who may be of normal weight but do not eat a balanced healthy diet. As such, the impact of DNA methylation on bodily processes has become of increasing concern as more research is conducted on dietary effects and the consequences of subsequent methylation patterns (overviewed in Figure 2).

The diet can affect DNA methylation due to the intake of choline, betaine, methionine, folic acid, and vitamins B2, B6, and B12, all of which are involved in the production of methionine [121,122]. These are primarily absorbed through diet rather that synthesised in the body. If a diet contains surplus of these molecules, then methionine will be increased. Methionine will then result in increased SAM production through the methionine cycle. SAM acts as a co-factor for the DNMT enzymes responsible in methylating DNA. Thus, a greater concentration of SAM results in a greater capacity to methylate DNA base pairs. Diets lacking methionine precursors will have an opposite effect, with reduced availability of methyl donors and methylation capability [122]. In practice, food contains many different compounds and therefore has differential effects on the methylation status of the genome. Thus, patterns and changes in the methylation of the genome have been the subject of recent investigation.

Diets which lack the precursors to the one-carbon cycle are likely to induce global methylation problems due to the inability to provide adequate molecules of SAM for DNMTs in order to transfer the methyl group, and convert SAM to SAH to cytosine residues. Such diets are similar to the methionine–choline deficient diets (MCD) commonly used in animal models. In studies investigating methylation, this usually includes folate deficiency as well (MCFD), or methyl–donor deficiency diets. When adolescent mice are fed an MCFD, it usually causes global hypermethylation to occur [123]. Within 3 weeks of being maintained on such a diet, mice were impaired in memory tasks, such as the novel object recognition task, as well as fear extinction [32]. Indeed, another study also found that developmental administration of MCFD resulted in impaired fear-associated memory lasting well into adulthood, although, surprisingly, anxiety was reduced [124].

### 3.1. Diet-Induced Methylation of the Dopaminergic System

The high fat (HF) diet has far-reaching effects regarding DNA methylation in the brain with the ability to affect the entire neurotransmitter systems. Vucetic et al. [125] found that chronic intake of a 60% fat diet in mice led to changes in the dopaminergic pathways. The ventral tegmental area (VTA), a key area in the pathway regulating reward behaviour, was found to be hypermethylated in the tyrosine hydroxylase dopamine transporter (DAT) as well as in the D1 and D2 receptor genes, whilst the hypothalamus, which is involved in homeostatic food signalling and intake, was hypomethylated. Both tyrosine hydroxylase and DAT are involved in the production and bioavailability of dopamine at the synapse. Therefore, changes in their epigenetics and genetic expression will affect dopamine levels. Indeed, Vucetic and colleagues report increased gene expression in the hypothalamus but decreased in the VTA, potentially providing a link as to why, in obesity models, anhedonia may be found, but excessive overeating is common.

Furthermore, the HF diet may cause overeating through addiction pathways similar those of to alcohol and nicotine. Addiction pathways overlap extensively with essential cognitive functions such as memory and impulse control, and are considered to be a disorder of altered cognition [126]. The HF diet may result in addiction like behaviours via the VTA, as well as D2 promotor hypermethylation within the striatum. This attenuates D2 receptor signalling, which, like a D2 receptor knockout rat model, induces addiction-like behaviours [34,127]. Thus, the differential methylation of dopaminergic genes drives an anhedonic response and a resulting compulsion to eat as a compensatory mechanism [127].

Indirect manipulation of the dopaminergic system is also caused by the methionine-choline–folate deficient diet (MCFD). Mice fed a MCFD diet had hypermethylated glutamate receptor 1 (GluR1) promotor regions resulting in reduced expression levels [32]. In knockout models of GluR1, mice consequently showed increased dopaminergic activity within the striatum of the brain, inducing cognitive and social deficits that were related to schizophrenia [128]. Moreover, GluR1 has been implicated in synaptic plasticity and long-term potentiation and therefore reductions in such may have deleterious effects upon spatial memory and cognition [129].

Similar to the MCFD, it is possible that a HF diet and resulting obesity may also cause schizophrenic-like cognitive deficits through dysfunctional dopaminergic signalling. However, it has only been ascertained that a maternal high fat diet increases risk of schizophrenia and neuropsychiatric conditions [130]. Whilst the dopaminergic system methylation status is dysregulated in schizophrenia, very little evidence suggests that in one lifetime, diet is sufficient to cause the disorder [131,132]. Given that DNA methylation can surpass the lifespan of one individual and pass to offspring, it is not inconceivable then that epigenetic causation of schizophrenia-like behaviours via a HF or MCFD diet requires more than a lifetime worth of predisposition, such that gross cognitive impairments require compounding in successive generations.

### 3.2. Dietary Effects on BDNF Methylation

The ability of the HF diet alone to impact cognition surpasses just dopaminergic systems. Indeed, HF diets can hypermethylate the BDNF gene and result in cognitive impairment [133,134]. Disruption of BDNF through methylation and, ultimately, reduced gene expression, has been associated with schizophrenia [135], cognitive impairments and hyperactivity [136,137].

BDNF remains a key factor throughout many dietary deficiencies. For example, zinc deficient diets result in hypermethylation at exon IX of the BDNF gene. This results in decreased expression of BDNF within the hippocampus and plasma of rats, as well as upregulation of DNMT1 and downregulation of DNMT3a. Because these are directly involved in methylation, disruption of DMNTs may cause global methylation patterns to change throughout the body [138]. Differential methylation of exon IX of BDNF (both hyper- and hypomethylation) has been found in sufferers of major depressive disorder in addition to reduced expression of BDNF [139]. The extent of the methylation of BDNF may also indicate the severity of symptoms seen in psychiatric problems [140]. Zinc deficiency results in methylation of exon IX. Moreover, zinc deficiency has been linked with treatment-resistant depression [141]. Therefore, it is possible that zinc is a major epigenetic regulator of depression and, through methylation, determines the success rate of other therapies. Preventing the methylation of BDNF in depressive patients, which may be caused by zinc (although not necessarily exclusively), via inhibition of DMNTs may also present a powerful method of treating symptoms. More research is needed in this area, but if zinc is influencing the efficacy of drugs, it may therefore be a suitable therapy for treatment-resistant sufferers [142].

Finally, diets which alter poly-unsaturated fatty acids (PUFA) status have been implicated in BDNF methylation as well as cognitive outcomes. A very common PUFA, Omega-3, reverses the hypermethylation of exon IV in BDNF caused by a high fat diet [143,144]. Indeed, upon supplementation of docosahexaenoic acid (DHA), the active ingredient in Omega-3, spatial learning and memory was improved, accompanied by increased BDNF and synaptic plasticity [145,146]. Moreover, disturbances within the PUFA biosynthesis gene Elovl5 are also associated with suicide attempt status [147]. The mechanism through which BDNF was found to be altered was the p38 mitogen activated protein kinase dependent mechanism [148] which is the same pathway that the HF diet may also activate via histone methylation [149]. Therefore, it is possible that PUFA deprivation and HF diets may share common mechanisms and may provide biochemical answers, through DNA methylation, as to why obesity is significantly associated with depression [150].

### 3.3. Diet and Methylation of Alzheimer’s Genes

Alzheimer’s disease (AD) is a neurodegenerative condition in which global methylation patterns have been seen to be altered [151]. The HF diet, as previously described, is a powerful modulator of the epigenome [152]. The HF diet is able to alter the methylation status of the cyclin-dependent kinase-5 (CDK5) gene, which, when translated to protein, is responsible in phosphorylating tau by binding to p35 and p25 proteins. The CDK5 promotor was hypomethylated by the HF diet in mice, which increased expression of CDK5 resulting in increased tau phosphorylation. This was accompanied with poorer performance in the Morris water maze test and novel object recognition, indicative of spatial learning and memory deficits [153].

The PPARα gene plays an important role in AD being a key modulator of secretase which in turns regulates the degradation of amyloid precursor protein B into amyloid B. Notably, when perturbed, this produces fibrils and plaques that are the hallmark of AD. Recent evidence indicates that diet can impact PPARα, and may therefore play a role in mediating Alzheimer’s onset or severity in those diagnosed. Carone et al., (2010) [154] fed mice a low protein diet which resulted in hypermethylation of a potential enhancer region of PPARα, resulting in reduced PPARα expression to levels seen in knockout models. Whilst this was specifically noted in the liver, diet can alter PPAR status in the brain [155]. This is of particular importance as PPARα deficiency inhibits neurodevelopment within zebrafish, and may therefore result in significant cognitive deficits in adulthood were it to be epigenetically silenced through diet [156]. Moreover, it may disrupt regulation of secretase in the adult brain and result in poorer clinical outcomes. Indeed, stimulation of PPARα activity in AD can reverse memory deficits and anxiety-like symptoms in genetic mouse models of AD [157].

Moreover, AD pathology also revolves around hyperphosphorylated tau. Folic acid has been found to reduce the phosphorylation of Tau in SH-SY5Y cells via methylation of the protein phosphatase 2 (PP2A) gene. This has been applied to type 2 diabetic mouse models induced by streptozotocin, which also significantly increased methylation of PP2A and the expression of DNMT1 mRNA, thereby suggesting that tau may be aggressively hyperphosphorylated upon folate deficiency [158,159]. Folate deficiency could also intensify or progress symptoms of AD through hypomethylation. The resultant effect is an increase in oxidative stress through increasing homocysteine levels, and whilst both oxidative stress and gradual hypomethylation are markers of Alzheimer’s, more work is needed to determine the severity of the effect of folate deficiency in adulthood on this disease [160].

To summarise, diets have a powerful modulatory effect on the epigenetic landscape and can impact entire neurotransmitter systems, such as dopamine, which may result in changes in reward and addiction habits (see Table 1 for a summary). Equally, the main detrimental effects of several diets may be attributed to a single molecule, such as BDNF that, when perturbed, has a significant effect on the cognitive performance of individuals and has been implicated in many disorders. And finally, diets, through epigenetic regulation, are able to contribute to the pathophysiology and outcomes of neurodegenerative disorders, highlighting that whilst diet may have a significant negative impact at any age, it stands to reason that diets can also be neuroprotective.

## 4. Neuroprotection through the Diet

An interesting aspect of the diet is the possibility to use food as a modulator of the epigenome. As we have previously described, the consumption of certain nutrients could modulate the interaction between gut and brain, not only leading to metabolism alteration but also to cognitive changes. Those observations would suggest that changes in the diet could induce epigenetic changes which ultimately would have an impact on cognitive functions. So, we will discuss the most promising results supporting the role of diet as a neuroprotective intervention.

### 4.1. Vitamins (Deficit/Supplement)

Vitamins from the diet can regulate both physiological and pathological processes through their impact on the epigenome [171]. The class of molecules that is most relevant in respect to its potential to modulate DNA methylation are the B vitamins: B12, B6, B9 (folate), and B4 (choline). These molecules are involved in the direct regulation of endogenous SAM (see Figure 1 for an overview). Once SAM becomes SAH, which is a competitive inhibitor of methyltransferases such as DNMTs, it is rapidly hydrolyzed to adenosine and homocysteine (Hcy). In the brain, Hcy is remethylated to methionine in a reaction requiring folate and B12 [172]. Thus, the brain is more prone to vitamin B deficiency. This deficiency could explain the local dyshomeostasis of the one-carbon metabolism in the brain, and of the consequent alteration of DNA methylation which may underpin several neurological and neurodegenerative diseases, such as amyotrophic lateral sclerosis, multiple sclerosis, Alzheimer’s disease, and Parkinson’s disease [173].

An example of the contribution of B vitamins to AD came from Fuso et al. [174] who fed transgenic mice containing a human mutated amyloid precursor protein (APP) a vitamin B-deficient diet. Their results showed increased DNA methylation, increased amyloid production, and greater cognitive impairment. Upon supplementation with the methyl donor SAM, all the AD-like features were restored to normality [166,167,175], revealing the contribution of vitamin B to the development of neurodegenerative disorders.

As we have previously discussed, B12 and folate deficits increase Hcy levels. Similarly, the presence of a B6 deficit increases Hcy levels. However, this comes about due to its mediation in the transformation from Hcy to cysteine. In this regard, several studies demonstrate that an intake of folic acid (0.5–5 mg/day) reduces Hcy in the blood by 25%, while this reduction increases up to 32% if combined with folic acid and vitamin B12 (0.5–5 mg day^−1^ and 500 µg day^−1^, respectively) [176,177]. The consequences of Hcy increase include oxidative stress, damage and alterations of DNA reparation, inhibition of methylation reactions, and, most importantly, neurotoxicity, which leads to cell death.

In line with this, Hcy seems to interact with the amyloid and tau pathway related to AD by inducing a Hcy-responsive endoplasmic reticulum stress protein, Herp, which interacts with Presenilin (PS) and increases the generation of amyloid-β (Aβ) [178]. It has also been shown that Hcy promotes tau hyperphosphorylation by inhibiting protein phosphatase 2 (PPA2) [179]. Thus, several studies have also linked Hcy to dementia, [180], depressive phenotypes [181] and Parkinson’s disease [182,183].

Moreover, it has been revealed that Hcy plays a role in cognitive impairment [184], such as a declines in episodic memory, semantic memory and global cognition [185], as illustrated by vitamin-induced changes in Hcy levels. Indeed, it has been shown that the supplementation with cobalamin (B12) improves cerebral and cognitive functions in the elderly [168]. In this study, the supplementation of cobalamin (1 month with placebo plus five months of treatment with an intramuscular injection of hydroxycobalamin (weekly 1000 µg for 4 weeks and monthly 1000 µg for 4 months) produced a decrease in Hcy and improved the scores in a verbal word learning test and verbal fluency. In another study, patients with cognitive impairment, defined as memory impairment or cognitive impairment insufficient for a diagnosis of dementia, with lower B12 levels, were supplemented by B12 [186]. The results showed that B12 supplementation in those patients significantly improved their verbal fluency, which reflects an improvement in frontal lobe function [169].

Regarding folate, its deficit has been associated with impairment in attention, episodic and visual spatial memory and abstract reasoning [163,164,165]. Indeed, the use of different folate supplements improved this cognitive impairment and had beneficial effects on dementia [187]. Similar results were found by Yuka Hama et al., (2020) who administered folate in deficient patients. They found that reduced Hcy levels correlated with better scores in the mini-mental state examination (MMSE) [188].

Finally, we would like to point out a study where a combination of different B vitamins were administered, including vitamin B12, B6 and folate. The results showed that the combined use of these vitamins have a positive effect on the reduction of Hcy concentration, thus, the daily doses of 5 mg folic acid, 1 mg B12, and 25 mg B6 for 18 months can reduce homocysteine levels by 26 % (from 9.2 to 6.78 mol/L) [189]. Another study observed a reduction of Hcy by 30.2% when using a similar cocktail of these vitamins (0.8 mg folic acid, 0.5 mg B12, 20 mg B6), which was paired with a reduction in brain atrophy by 29.6% when compared to the placebo group. Although we are highlighting the most common vitamin deficits associated with current diets, it is important to note that other B vitamins affect brain function, as a deficit in B1-B21 could result in disturbed sleep and memory loss; deficiency in B2 could cause brain dysfunction; B3 was linked to depression and anxiety; encephalopathy, behavior change, demyelination were associated to B5; and lethargy, hallucinations and seizures were found when there was a deficiency in B7 [181].

### 4.2. Choline (Deficit/Supplement)

Another important nutrient that should be considered when using diet as a neuroprotector is choline. This nutrient is present in eggs, wheat germ, chicken, and beans among others, and together with folate, vitamins B6 and B12, and methionine participates in DNA methylation. Indeed, methionine, as described previously, could come from the methylation of Hcy by methyltetrahydrofolate, or through a methyl group derived from betaine obtained through the oxidation of its precursor choline. These choline-containing compounds are all interchangeable within the body, but the conversion of choline to betaine is irreversible [190]. So, choline deficits are related to increased Hcy [191] as illustrated by da Costa et al. [192] who observed that human and mice under a diet low in choline have difficulties in removing Hcy, leading to elevated concentration in the plasma. In this regard, an increased intake in choline in animal models of Down syndrome has shown a positive cognitive effect in several visual attention tasks, affective and neural functioning and the normalization of hippocampal neurogenesis [193,194,195].

Moreover, choline intake has been related to several neurodegenerative and neuropsychiatric disorders. Stevens et al. studied the impact of prenatal choline intake in a deficient sensory inhibition animal model which mimics schizophrenia. Their results proved that animals with prenatal choline supplementation had normal sensory processing, concluding that choline may have an important role in reducing schizophrenia-like symptoms [196]. Similar results were observed in a mouse model of AD, which corroborated AD’s propensity for memory problems as a result of degeneration or dysfunction of the septo-hippocampal cholinergic neurons [197]. Choline supplementation in this mouse model seems to prevent degeneration of these neurons and improve AD symptoms [161]. Also, Mapstone et al. showed that AD patients have lower concentration of different choline forms compared with healthy controls. Indeed, these changes have been validated as a predictor of AD with an accuracy of 90% [198], highlighting the need to control this nutrient in our diets.

However, contradictory results were found in adult humans where no differences in cognition after choline intake were observed [199,200,201]. Contrary to those results, Nurk et al. found that better scores on test for sensorimotor speed, executive function and perceptual speed were related to higher blood choline levels [162]. In sum, despite the correlation between choline availability and improved cognition, more studies are needed to shed light on the mechanism behind its positive impact.

### 4.3. Methionine (Deficit/Supplement)

Methionine can be ingested by the consumption of lean meat and eggs, although lower quantities can be found in plant sources. It is worthy to mention the close interrelationship of choline, folate, vitamin B12, and methionine. Their metabolism intersects at the formation of methionine from homocysteine. Methionine can be formed through two pathways, as mentioned previously, so a disturbance in one of these metabolic pathways results in compensatory changes in the other. Surprisingly, however, it has been suggested that dietary methionine restriction (MR) could extend the life span by increasing energy intake and expenditure without food restriction.

In line with this, studies coming from Orentreich group have shown that reducing dietary methionine was able to increase the longevity of the rats without food restriction [202,203]. The effects caused by the methionine restriction in the diet were originally linked to the availability of cysteine, which is the limiting amino acid in glutathione (GSH) synthesis [204]. GSH is a tripeptide that has received considerable attention as a possible protective agent against oxidative damage. The decline in the availability of this important antioxidant and detoxifying reagent has been linked to age-related decline. However, the results from Zimmerman et al., (2003) [205] were contradictory results in this respect. Indeed, similar results were found in different species such as mice and flies [206,207,208], proving the consistency of the data across species.

Longevity linked to methionine metabolism has been also linked to the reversed oxidation of methionine by the methionine sulfoxide reductases A (MsrA) in flies [209]. Moreover, targeted disruption of MsrA in mice significantly reduced their life span, increased their susceptibility to oxidative stress, and accentuated the accumulation of oxidized proteins in their tissues [202].

Regarding the data, some questions need to be addressed in future studies, such as how dietary restrictions could influence essential amino acid (EAA) absorption by the gut, how those EAAs impact the brain, and how changes in EAAs would affect gut–brain communication, influencing cognitive processes.

### 4.4. Probiotics

The effect of probiotics on cognition is a novel field we would like to approach in this review. Several studies have shown that the gut and the brain are bidirectionally connected by several pathways, including the neural, immune, metabolic, and endocrine pathways [210,211,212]. The gut microbiota could be an environmental factor influencing brain epigenetics and modulating brain function [84].

Numerous investigations have proven that the composition of the gut microbiota has a direct relationship with normal brain function. An imbalance in gut microbiota composition has been associated with many brain diseases in which cognitive dysfunction is a common clinical problem (i.e., neurodevelopmental, psychiatric and neurodegenerative disorders) [213]. Many studies have tried to explain the routes connecting brain epigenetics and gut microbiota, and it has been shown that this bidirectional relationship is subject to direct and indirect manipulation. Some products of bacterial metabolism, such as butyrate and propionate, are well known key modulators of several epigenetic erasers, such as HDACs [214,215]. Furthermore, DNMTs, the enzymes catalysing the transfer of a methyl group on a CpG dimer, are highly sensitive to the availability of SAM. SAM levels may be, in turn, regulated by the abundance of some molecules, including folate, which supports the one-carbon metabolism also provided by gut microbial communities [94,216,217,218]. Cuomo and colleagues [76] proved that change in microbiota composition affected brain epigenetics leading to long-lasting effects on specific brain gene regulation in Zebrafish (*Danio rerio*). They investigated the methylation state of the BDNF and Tph1A promoter region in the brain and gut of probiotic-treated and untreated zebrafish and found slight DNA methylation changes in probiotic-treated samples indicating that specific DNA methylation signatures significantly correlated with specific behavioural scores.

Several studies have also found that a probiotic supplement positively affects cognition in control animals. For example, Savignac et al., (2014) found that *Bifidobacterium longum* improved cognitive function in healthy Balb/c mice, as measured by the novel object recognition task and the Barnes maze tests [219]. Moreover, Bravo et al., (2011) reported that *Lactobacillus rhamnosus* increased memory consolidation in stress-induced hyperthermia when compared to controls as well as reducing depression/anxiety-like behaviours as measured by the forced swim test, and the elevated plus maze [62]. Also, it has been found that *Bifidobacterium longum* 1714 substantially enhanced the learning and memory capabilities evaluated by the fear conditioning test, the novel object recognition task, and the Barnes maze test [219].

The favourable effect of probiotics on cognitive function in healthy humans is also considered in several studies. Messaoudi and colleagues (2011b) found that the administration of *Lactobacillus helveticus* strain R0052 and *Bifidobacterium longum* strain R0175 showed beneficial impact on overall cognitive function, which was measured using a questionnaire assessing problem-solving strategies in healthy adult populations [220]. Another experiment has shown that the intake of *Bifidobacterium longum* 1714 in healthy male volunteers exposed to acute stress improved hippocampus-dependent visuospatial memory performance [221].

Apart from the studies which show the positive effects of probiotics on cognition in healthy subjects, several studies have been conducted in animal models of disease. For example, Liang et al., (2015) found that *Lactobacillus helveticus* NS8 administered to rats under chronic restraint stress improved object novelty detection and object location memory [222]. Furthermore, rats administered with the probiotic *Lactobacillus helveticus* strain NS8 in a model of hyperammonaemia-induced neuroinflammation showed significant restoration of cognitive function, reductions in inflammatory markers, and improved anxiety-like behaviour [223].

Although less investigations have been carried out on human subjects, several studies have searched for a link between gut microbiota and neurological disorders. In this sense, it has been shown that gut microbial metabolites may increase or decrease the risk of AD [224,225]. Moreover, some other brain diseases, including neurodegenerative and neurobehavioral disorders such as multiple sclerosis [226,227], Parkinson’s disease [228,229] and autism spectrum disorder [230,231] are also subjected to altered gut microbiota, and the positive effects of probiotics on behavioural performance have been described.

Studies in animals show that microbiota and probiotics may have an important role in animal welfare and could be related to the development of different diseases. For example, Borre et al., showed how the disruption of microbiota in mice induced anxiety, depression and even autism [232]. The same author reported that Bifidobacterium seems to be more effective than Lexapro, a drug used to treat anxiety and depressive behaviours [219]. In the same line, in humans, regular intake of *Lactobacillus helveticus* and *Bifidobacterium longum* for 30 days reduces stress levels and depression scores in healthy humans [233]. Another study linked the intake of yogurt or multi probiotics with mental health [234] and even improvements in mindset in those suffering from poor mood.

Finally, in patients with AD or depression, probiotics have been shown to improve cognitive deficits [235]. Indeed, consumption of multi probiotics (*Lactobacillus acidophilus, Lactobacillus casei, Bifidobacterium bifidum* and *Lactobacillus fermentum*) for 12 weeks improved metabolic status and cognitive function in patients with AD [235]. In this study, AD patients who drank 200mL of milk with the aforementioned probiotics showed a significant improvement in MMSE score compared to the control group. Another study showed that the administration of multiple probiotics such as *Lactobacillus acidophilus, Bifidobacterium bifidum, Bifidobacterium longum* in combination with selenium for 12 weeks were able to improve cognitive functions (higher MMSE score) in patients with AD [236]. In depression, probiotic intake for eight weeks seems to improve depression inventor scores [237].

In sum, the benefits of probiotics on cognition have been demonstrated in both animals and humans. Moreover, their positive effect in humans has been shown not only in healthy subjects but also under different disease conditions such as AD, depression and subclinical poor mood. However, further studies are needed to understand the environmental changes driving microbiota perturbation, which ultimately effects epigenetic responses. Furthermore, the mechanisms behind the epigenome’s role in the microbiota–gut–brain axis would require further investigation in order to develop a microbiota-based therapy as an alternative pathway to treat brain disorders.

### 4.5. Diets

As previously described, different nutrients from the diet could have a significant impact on cognition. In this section we will discuss some general diets and how they could be related to brain changes. Firstly, we would like to approach the vegetarian or vegan diet where there is no meat intake. These diets are lacking vitamin B12, although folate intake is higher than an omnivorous diet. In this regard, Gilsing et al., demonstrated that omnivorous diets have the highest (270–292 pmol/L) vitamin B12 levels compared to vegetarian (175–189 pmol/L) or vegan diets, which have the lowest amount of vitamin B12 (117–127 pmol/L). Indeed, 52% of vegans and 7% of vegetarians who participated in the study were classified as B12-deficient. Opposite results were found in folate concentrations, where vegans had higher levels than vegetarians, whilst omnivores had the lowest levels. Surprisingly, two people (omnivores) were categorized as folate deficient [238].

Moreover, a vegetarian diet was associated with lower scores in the depression anxiety stress scale (DASS), and profile of mood state (POMS) questionnaires [239]. Beezhold’s group found that mood scores significantly improve after two weeks with a vegetarian diet [240], whereas omnivores’ stress levels are lower than vegans’ [241]. Contrary to these data, Baines et al., have found that vegetarian women have poorer mental health, which is associated with increased depression compared to non-vegetarian women [242]. Another study comparing 100 vegetarians and 100 omnivores found that the development of depression in the vegetarian group was the highest (31% vs. 12%). The same tendency was also observed for paresthesia (11% vs. 3%), peripheral neuropathy (9% vs. 2%), and psychosis (11% vs. 3%). All these findings correlated with a decreased vitamin B12 concentration in the vegetarian group’s plasma [170]. These results illustrate a key controversy in the field, where several studies show a positive relation between vegetarian and vegan diets with mental health and cognition [239,243], whereas the opposite effect is reported in others [170,242,244,245].

Extremely important for this review is the Mediterranean diet, which is characterized by high intake of fruits, vegetable, cereal, legumes and olive oil, with low intake of saturated fats, moderate intake of fish and alcohol (especially wine), and low consumption of meat products [246]. The Mediterranean diet contains high levels of folate, B6 and B12 vitamins and choline, and it contains different probiotics derived from milk products [247]. It has been demonstrated that individual components of the Mediterranean diet and daily adherence to it reduces oxidative stress biomarkers and positively impacts cognition [247]. Indeed, adherence to a Mediterranean diet has an inverse association with the Hcy levels that, as we previously discussed, seem to have a neuroprotective effect. In line with this, Foscolou et al., measured Hcy levels in subjects with low, moderate and high adherence to the Mediterranean diet. Their findings showed that lower Hcy levels were found in subjects with moderate or high adherence [248]. Thus, the Mediterranean diet may reduce depression, cognitive decline [249], risk of dementia, AD [250,251,252] and predementia syndrome (which refers to all conditions with age-related deficits in cognitive function, including a mild stage of cognitive impairment based on a normality model and pathological conditions considered predictive of early stages of dementia) [253]. Other studies also showed an association between adherence to the Mediterranean diet, a reduction in risk of mild cognitive impairment (non-demented aged persons with no significant disability and a mild memory or cognitive impairment which cannot be explained by any recognized medical or psychiatric condition) and a reduction in the progression of mild memory or cognitive impairment in AD [254].

Apart from the beneficial effects of B vitamins and choline present in the Mediterranean diet on DNA methylation, as we have previously discussed, we would like to note the specific contribution of olive oil. Indeed, the Mediterranean diet has a high concentration of unsaturated fatty acids, such as olive oil [255,256]. In this regard, different studies have demonstrated how the intake of unsaturated fatty acids is related with an improvement in cognitive functions measured through the MMSE (mini-mental state examination) [257,258].

Finally, another component of the Mediterranean diet that may have a positive effect on cognition is the red wine which is consumed in moderate amounts [246]. The effect on cognition could be due to the polyphenols present in the red wine [259]. Thus, polyphenols have the capacity to modulate and equilibrate the gut microbiota [260]. This positive regulation of gut microbiota may have a positive effect on cognition, as previously described.

## 5. Conclusions and Future Directions

Different types of diets such as ketogenic, very low carbohydrate, high fat, adequate protein, and caloric restricted diets, among others, could have an impact on the metabolic status of the brain. That connection does not only rely direct impacts, but also on the connection between the gut microbiota and the brain. Moreover, several studies have shown the connection between nutritional challenges and DNA methylation in the brain during adulthood, although the mechanisms behind how macro- or micronutrients from the diet could impinge on neural epigenetic modifications needs further investigation.

Moreover, important questions remain, such as the existence of brain-sensitive areas to DNA methylation and their connection to changes in cognition, the possibility to predict neurodegenerative diseases based on the brain DNA methylation profile, and the specific role of gut microbiota and its metabolites in brain DNA methylation.

Another emerging field is the role of diets as an epigenetic modifier of the genome by changing the availability of methyl donors such as SAM. SAM synthesis depends on dietary ingestion and modulates neuronal gene expression and brain function. Therefore, the identification and implementation of effective nutritional strategies could improve cognition and mental health.

Therefore, the study of the relationship between gut microbiome and brain may significantly enhance our understanding of nutrient metabolism and specific pathways by which diet can influence health and cognition. This may facilitate the adoption of an individualized, nutrition-based approach to target gut microbial structure and function, as well as the potential to alter the availability of different metabolites in the brain.

## Figures and Tables

**Table 1:** Different dietary regimes and their effects on DNA methylation and the resulting changes in the brain. Abbreviations: AMPA, amino-3-hydroxy-5-methyl-4-isoxazolepropionic acid receptor; GluR1, glutamate receptor 1; DA, dopamine; LTP, long-term potentiation; AD, Alzheimer’s disease; PP2A, protein phosphatase; Hcy, homocysteine; DNMT, DNA methyltransferase; DAT, dopamine active transporter; D1R/D2R, dopamine 1/2 receptor; BDNF, brain-derived neurotrophic factor; CDK5, cyclin-dependent kinase 5; Tph1A, tryptophan 5-monooxygenase; PPARα, peroxisome proliferator-activated receptor alpha.

Diet/Supplementation	Changes in Methylation Patterns	Physiological and Behavioural Effects
MCD/MCFD diet	○ Global hypermethylation [123] ○ Specific methylation of AMPA subunit promotors and GluR1 [32]	Impaired fear extinction [124] and hippocampal-dependent memory [32]. Reduction in GluR1 expression, which increased DA levels in the striatum, resulting in schizophrenia-like behaviours. Reductions in LTP and deficits in synaptic plasticity [32,128,129].
Supplementation of Methionine/Choline	○ Global DNA hypermethylation [33]	Ameliorated depressive-like symptoms resulting from maternal deprivation in rats [33]. Choline improved visual attention tasks/affective neural functioning and hippocampal neurogenesis. Evidence to suggest it may mitigate the pathogenesis of schizophrenia/AD [161,162].
Choline Deficiency	○ Changes in global methylation patterns [100]	Learning and memory deficits [100].
Folate Deficiency	○ Hypomethylation [160] ○ Reduced methylation of PP2A gene [158,159]	Cognitive impairment [96], increased Hcy and oxidative stress [160]. Impaired abstract reasoning, visuospatial memory and attention [163,164,165] Hyperphosphorylated tau (associated with AD) and increased DNMT1 mRNA [158,159].
High Fat Diet	○ Global DNA hypermethylation [127,135,136,137] ○ Hypermethylation of dopaminergic genes: DAT, tyrosine hydroxylase, D1R and D2R [34,125] ○ Hypermethylation of BDNF [133,134] ○ Hypomethylation of dopaminergic genes in hypothalamus [125] ○ Hypomethylation of CDK5 [153]	Hypermethylation: Downregulation of D2Rs in the striatum (rescued by oryzanol) [34] and the VTA, which was reported to give rise to anhedonia and addiction-like behaviours [125,127], as well as exacerbation of schizophrenia symptoms [135], hyperactivity and cognitive impairment [136,137]. Hypomethylation: increased CDK5 expression and resulting tau hyperphosphorylation. Followed by memory impairments in mice [153]. Increase of DRs in hypothalamus resulting in compulsive eating [125].
SCFAs	○ Reversal of exon IV BDNF hypermethylation (Omega-3) [143,144]	Increased BDNF expression, and improvement in spatial learning and memory [145,146]. Dysregulation associated with suicide status via p38 mitogen kinase pathway [147,148].
Caloric Restriction	○ Changes in the pattern of CG and CH methylation [36]	Improved cognition and neurogenesis in mice [36]
Probiotics	○ Global changes in DNA methylation patterns including BDNF and Tph1A [76]	Improved behaviour, mood and cognition [76,77,78]
Low Protein	○ Hypermethylation of (potential) PPARα enhancer [154]	Reductions in PPARα increase amyloid fibrils (associated with AD) [154]. There is evidence to suggest that an increase in PPARα improves memory and anxiety in AD mice [157].
Zinc Deficiency	○ BDNF exon IX hypermethylation	Decreased expression of BDNF and DMNT3a and upregulation of DNMT1 [138]. Implicated in depression, particularly treatment-resistant [139,142].
Vitamin B		
Pan-vitamin B Supplementation	○ Hypermethylation of Redox Genes [119]	Improved cognitive performance [119]
Vitamin B2 Supplement (Riboflavin)	○ Unexplored	Improved global cognitive function in elderly human subjects [99]
Vitamin B6 Deficiency	○ Hypomethylation [reviewed in [166]	Increased brain levels of Hcy, exacerbating AD pathology and symptoms of cognitive impairment [167].
Vitamin B12 Supplement	○ Unexplored	Improved cognition, decreased Hcy [168,169]
Vitamin B12 Deficiency	○ Unexplored	Correlates with paresthesia, peripheral neuropathy and psychosis in vegetarians [170].

## Abbreviations

AD	Alzheimer’s disease
BBB	Blood–brain barrier
BDNF	Brain-derived neurotrophic factor
CNS	Central nervous system
DNMT	DNA methyltransferase
ENS	Enteric nervous system
Hcy	Homocysteine
HDAC	Histone deacetylase
HF	High fat diet
LTP	Long-term potentiation
MCD/MCFD	Methionine-choline (folate) deficient diet
MMSE	Mini-mental state examination
PUFA	Poly-unsaturated fatty acids
SAM	S-adenosyl methionine
SAH	S-adenosyl homocysteine
SCFA	Short-chain fatty acids
